# Mutation spectrum of *EXT1* and *EXT2* in the Saudi patients with hereditary multiple exostoses

**DOI:** 10.1186/s13023-021-01738-z

**Published:** 2021-02-25

**Authors:** Zayed Al-Zayed, Roua A. Al-Rijjal, Lamya Al-Ghofaili, Huda A. BinEssa, Rajeev Pant, Anwar Alrabiah, Thamer Al-Hussainan, Minjing Zou, Brian F. Meyer, Yufei Shi

**Affiliations:** 1 Department of Orthopedics, King Faisal Specialist Hospital and Research Center, Riyadh, Saudi Arabia; 2 College of Medicine, Alfaisal University, Riyadh, Saudi Arabia; 3 Department of Genetics, MBC 3, Centre for Genomic Medicine, King Faisal Specialist Hospital and Research Center, P.O. Box 3354, Riyadh, 11211 Saudi Arabia

**Keywords:** *EXT1*, *EXT12*, Mutation, Exostoses, Osteochondromas

## Abstract

**Background:**

Hereditary Multiple Exostoses (HME), also known as Multiple Osteochondromas (MO) is a rare genetic disorder characterized by multiple benign cartilaginous bone tumors, which are caused by mutations in the genes for exostosin glycosyltransferase 1 (*EXT1*) and exostosin glycosyltransferase 2 (*EXT2*). The genetic defects have not been studied in the Saudi patients.

**Aim of study:**

We investigated mutation spectrum of *EXT1* and *EXT2* in 22 patients from 17 unrelated families.

**Methods:**

Genomic DNA was extracted from peripheral leucocytes. The coding regions and intron–exon boundaries of both *EXT1* and *EXT2* genes were screened for mutations by PCR-sequencing analysis. Gross deletions were analyzed by MLPA analysis.

**Results:**

*EXT1* mutations were detected in 6 families (35%) and 3 were novel mutations: c.739G > T (p. E247*), c.1319delG (p.R440Lfs*4), and c.1786delA (p.S596Afs*25). *EXT2* mutations were detected in 7 families (41%) and 3 were novel mutations: c.541delG (p.D181Ifs*89), c.583delG (p.G195Vfs*75), and a gross deletion of approximately 10 kb including promoter and exon 1. Five patients from different families had no family history and carried de novo mutations (29%, 5/17). No *EXT1* and *EXT2* mutations were found in the remaining four families. In total, *EXT1* and *EXT2* mutations were found in 77% (13/17) of Saudi HME patients.

**Conclusion:**

*EXT1* and *EXT2* mutations contribute significantly to the pathogenesis of HME in the Saudi population. In contrast to high mutation rate in *EXT 1* (65%) and low mutation rate in *EXT2* (25%) in other populations, the frequency of *EXT2* mutations are much higher (41%) and comparable to that of *EXT1* among Saudi patients. De novo mutations are also common and the six novel *EXT1*/*EXT2* mutations further expands the mutation spectrum of HME.

## Introduction

Hereditary Multiple Exostoses (HME) or Multiple Osteochondromas (MO) is a rare autosomal-dominant pediatric disorder with an incidence of about 1 in 50,000 individuals and male-to-female ratio of about 1.5:1 [1, 2]. The disease is characterized by the development of two or more cartilage capped bony outgrowths within perichondrium in long bones and ribs, which can cause a variety of orthopedic deformities such as disproportionate short stature, shortened forearms, and unequal limb length. Although it is generally a benign skeletal tumor, 2.8% (0.5–5%) of patients undergo malignant transformation towards life-threatening chondrosarcomas or osteosarcomas due to their typical resistance to chemo- or radiation therapy [3, 4].

Germline heterozygous loss-of-function mutations in the *EXT1* (exostosin-1, located on chromosome 8q23-q24) or *EXT2* (exostosin-2, located on chromosome 11p11-p12) tumor suppressor genes are responsible for over 70–95% of HME cases [5, 6]. There are 566 *EXT1* and 278 *EXT2* mutations reported in the literature (HGMD database). The majority of these mutations (79% in *EXT1* and 75% in *EXT2*) are frameshift, nonsense, and splice-site mutations, resulting in truncated proteins [5]. About 65% of the mutations occur in *EXT1* and 25% in *EXT2*. In about 10–15% of HME cases, genomic alterations cannot be detected by the conventional method due to alterations such as intronic deletions, translocations or somatic mosaicism [7, 8]. The involvement of other genes or the putative *EXT3* gene on chromosome 19 still needs investigation.

The genetic defects causing HME have not been systematically investigated in the Arab population. In the present study, we performed molecular analysis of 22 patients from 17 unrelated Saudi families with HME. *EXT1* or *EXT2* mutations were identified in 77% of patients (13/17) including six novel mutations.

## Subjects and methods

### Patients

**Fig. 1:**
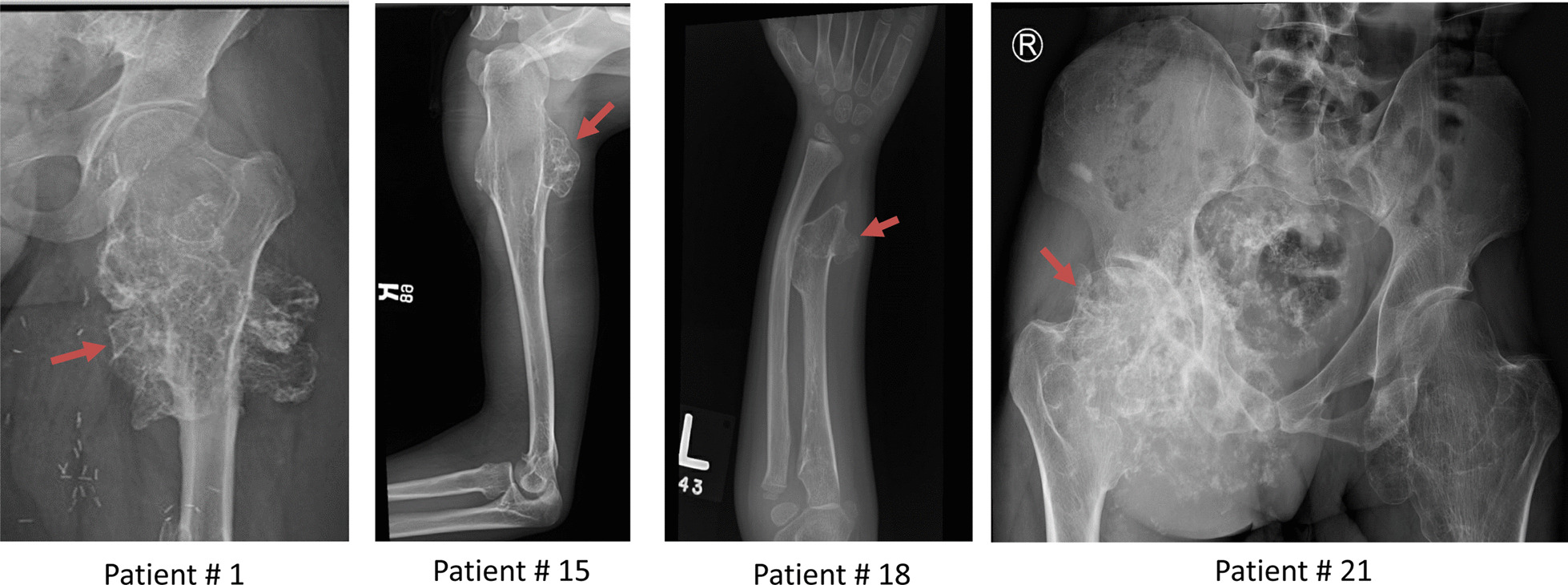
Radiology of patients with osteochondromas. Patient#1 has an osteochondroma at left hip joint; Patient #15 has an osteochondroma at right proximal humerus; Patient#18 has an osteochondroma at left distal radius; and Patient # 21 has a right pelvic osteochondroma with malignant transformation. Osteochondroma is indicated by an arrow

Seventeen Saudi families with HME were investigated (Fig. 1 and Table 1). The inclusion criteria were two or more exostoses diagnosed upon physical and radiographic examinations. Disease severity was divided into 3 classes based on the presence of skeletal deformities and functional limitations using the following criteria: Class I: no deformities and no functional limitations [A ≤ 5 sites with osteochondromas, B > 5 sites with osteochondromas]; Class II: deformities and no functional limitations [A ≤ 5 sites with deformities, B > 5 sites with deformities]; and Class III: deformities and functional limitations [A functional limitation of 1 site, B functional limitation of > 1 site] [9]. Blood samples were obtained from patients and available relatives for genomic DNA extraction after informed consent. The study was approved by the Ethics Committee of King Faisal Specialist Hospital and Research Centre (RAC # 2170 027). Written consent was obtained from the patients or guardian of the patients before enrollment.

**Table 1:** Genetic defects in 17 families with hereditary multiple Exostoses

Family	Pt #	DX Age (year) and Height (cm)	Onset Age (year)	Sex	Clinical features	EXT1 mutation NM_000127	EXT2 mutation NM_207122	Family history
1	1	32, 157	18	M	Class IIIB severity and surgical excision of exostosis from left hip joint duo to decreased range of motion and pain. Two exostoses were found	c.1469delT (p.L490Rfs*9) exon 6, reported [37]	ND	Younger sister is affected
2	2	17, 156	8	M	Class IIIB severity and surgical excision of exostosis from the head of fibula duo to nerve compression. Four exostoses were found	#c.739G > T (p. E247*), exon 1, novel mutation (not reported in the literature)	ND	NO
3	3	18, 149	8	M	Class IIA severity and surgical excision of left distal tibia exostosis excision. Two exostoses were found	ND	ND	NO
4	4 Father	40, 165	N/A	M	Class IA severity (mild asymptomatic disease). Two exostoses were found	ND	c.626 + 2_626 + 5delTAGG, intron 3, reported [38]	
	5	14, 149	10	F	Class IIIB severity and surgical excision of two exostoses on the right leg	ND	c.626 + 2_626 + 5delTAGG, intron 3, reported [38]	2 siblings are affected
5	6	18, 149	11	F	Class IIIB severity and surgical excision of left proximal femur and tibia exostoses. Three exostoses were found	#c.1319delG (p.R440Lfs*4), exon 5, novel mutation	ND	NO
6	7 Father	55, 164	N/A		Class IA severity (mild asymptomatic disease). Two exostoses were found	ND	c.541delG (p.D181Ifs*89), exon 3, novel mutation	
	8	29, 156	10	F	Class IIA severity with mental retardation, epilepsy and developmental disorder Two exostoses were found	ND	c.541delG (D181Ifs*89), exon 3, novel mutation	5 siblings are affected
	9	9, 133	7	M	Class IA severity (mild asymptomatic disease). Two exostoses were found	ND	c.541delG (D181Ifs*89), exon 3, novel mutation	
7	10	24, 180	18	M	Class IA severity (mild asymptomatic disease). Two exostoses were found at distal right femur	ND	c.544C > T, p.R182*, exon 3, reported [39]	All of his 5 brother and 4 of 7 sisters are affected
8	11	23, 160	10	F	Class IA severity (mild asymptomatic disease). Four exostoses were found	ND	# 10 kb homozygous deletion including promoter and exon 1, novel mutation	NO
9	12 Mother	62, 156	N/A	F	Class IA severity (mild asymptomatic disease). Two exostoses were found	ND	homozygous c.540G > A (p.W180*), exon 3, reported [11]	Her brother is affected with no symptoms
	13	36, 160	15	F	Class IIIB severity and surgical excision of exostoses and deformity correction. Three exostoses were found	ND	c.540G > A (p.W180*), exon 3, reported [11]	
10	14 Father	49, 165	N/A	M	Class IA severity (mild asymptomatic disease). Two exostoses were found	c.1021A > G, (p.R341G), exon 2, reported [5]	ND	
	15	23, 170	18	M	Class IIIB severity and surgical excision of exostoses from both tibia, femur, and radius. Currently complaining of left hip pain. Three exostoses were found	c.1021A > G (p.R341G), exon 2, reported [5]		Yes, all of his 4 brothers and one sister are affected with mild form of the disease
11	16	22, 171	18	F	Class IIIA severity and surgical excision of exostoses from head of the fibula. Three exostoses were found	ND	ND	NO
12	17	29, 162	10	M	Class IIIB severity and surgical excision of exostoses from right proximal tibia, right distal tibia and 4^th^ rib excision. Three exostoses were found	ND	ND	NO
13	18	15, 159	10	F	Class IIIA severity. Two exostoses were found in the upper extremities	ND	ND	NO
14	Father	42		M	normal	ND	ND	
	Mother	41		F	normal	ND	ND	
	19	15, 140	6	M	Class IIIB severity and surgical excision of exostosis from left hip joint duo to decreased range of motion and pain. Four exostoses were found	#c.1786delA, (p.S596Afs*25, exon 9, novel mutation	ND	NO
15	20	29, 169	8	M	Class IIIB severity and surgical excision of exostoses from left and right knees. Five exostoses were found	ND	#c.484delC (p.Q162Rfs*108), exon 2, reported [40]	NO
16	21	42, 170	20	M	Class IIIB severity with malignant transformation to osteochondrosarcoma at right pelvic. Three exostoses were found	ND	c.583delG (p.G195Vfs*75), exon 3, novel mutation	Yes, several nephews of his are affected but none of them required clinical intervention
17	22	7, 120	7	M	Class IIA severity with symptomatic bone deformities. Two exostoses were found	^#^heterozygous deletion of exon 2–11, reported [41]	ND	NO

ND: not detected; #de novo mutations. Disease severity is divided into 3 classes using the following criteria: Class I: no deformities and no functional limitations [A ≤ 5 sites with osteochondromas, B > 5 sites with osteochondromas]; Class II: deformities and no functional limitations [A ≤ 5 sites with deformities, B > 5 sites with deformities]; and Class III: deformities and functional limitations [A functional limitation of 1 site, B functional limitation of > 1 site] [9]

### Genomic DNA isolation

Genomic DNA from peripheral blood leukocytes was extracted as described previously [10].

### DNA amplification and sequencing

DNA samples were analyzed for mutations in all the coding exons and intron–exon boundaries of *EXT1* and *EXT2* genes by polymerase chain reaction (PCR) and sequencing analysis*.* PCR primers and conditions were described previously and listed in Table 2 [11]. The resulting PCR products were directly sequenced with BigDye Terminator 3.1 Cycle Sequencing kit using an automated ABI PRISM 3700 sequencer (Applied Biosystems; Life Technologies, Foster City, CA).

**Table 2:** *EXT1* and *EXT2* primer sequences and PCR conditions

Exons	*EXT1*-Forward	*EXT1*-Reverse	Annealing ( ºC)	*EXT2*-Forward	*EXT2*-Reverse	Annealing (ºC)
Exon 1a	5′ggaaaggcatccagagaaggt-3’	5′-cttgcaaagggtgaaatcgaa-3’	58	5′-cagtccgctccttcctttcct-3’	5′-agtgcctggcccaacatgac-3	62
Exon 1b	5′-ttcgttccttgggatcaatt-3’	5′-cctgtcctgggatgatcctta-3’	56			
Exon 1c	5′-ggcacttggcctgactacac-3’	5′-gggctcatccgccctcacc-3’	58			
Exon 2	5′-gagttgctttgcgtaaattca-3’	5′-acaccttctctttagctatcc-3’	58	5′-aggttgaatagtcttttcaag-3’	5′-ggaaaccaactcaagagcagaa-3’	54
Exon 3	5′-cagtcattgagtttgtactga-3’	5′-gagctgaccttttggattcat-3’	58	5′-ggatccttgatagttgttgtc-3’	5′-caattctgattacaaagtatg-3’	58
Exon 4	5′-ctatatgctagaagccaaatg-3’	5′-cactggaccaatcacacatcc-3’	56	5′-gactcagtaattcctgttcct-3’	5′-gcctcaaggaccctaccctg-3’	56
Exon 5	5′-gtcactactctgactgccacc-3’	5′-tgcagggtgttagatggacc-3’	58	5′-ctggtaaggaaacacttactg-3’	5′-ctagttgcatgctgaaaacaa-3’	58
Exon 6	5′-ctccagcatgaggcagcggag-3’	5′-gggtatgatgttagagaagt-3’	58	5′-cagtattgcttggcgtcaacc-3’	5′-tgtagtagttcttgaaccagg-3’	58
Exon 7	5′-ctctttctgtctctgagaaga-3’	5′-gaacagggagaagatatctag-3’	58	5′-gatgttgtttctgcttgtgaa-3’	5′-gatctagtggaggaagtaaac-3’	56
Exon 8	5′-caggtgaggatgggagaattg-3’	5′-gaagcattagcatcgtgcaac-3’	58	5′-aaaggaattagcctaacctgg-3’	5′-cctttacaattgtagtacatt-3’	58
Exon 9	5′-gaattaatgtttcgccacagt-3’	5′-ctgttaacaagatttggcctt-3’	58	5′-caccaagcctgccatgtttgg-3’	5′-ggtattgctattgacaaagca-3’	58
Exon 10	5′-gacatgtttagggattcaaag-3’	5′-ctcctcattatatgctcctgg-3’	58	5′-gctgattctcccatctcattt-3’	5′-ttacgcacaccttttggactc-3’	58
Exon 11	5′-gctgcttgctcatttgcctg-3’	5′-caggagttgagttctcattgg-3’	58	5′-gatggtttgaacctaggaagt-3’	5′-ctaagccctcttggcaggtat-3’	58
Exon 12				5′-ccatgccttggctatgctgcc-3’	5′-gttacaagaacttcctaggct-3’	58
Exon 13				5′-caacatctcagcttacaacac-3’	5′-tatggctaccagctgctgtcc-3’	58
Exon 14				5′-ctctcaacctcttgaacatac-3’	5′-gtgcatgccaagatccaagta-3’	58

PCR conditions: 50 ng of DNA were denatured at 95 °C for 5 min on initial cycle followed by 35 cycles of denaturation, annealing, and extension at 1 min on each step

### Analysis of copy number variation

Copy number variation in genomic DNA was analyzed by MLPA (Multiplex Ligation-dependent Probe Amplification) analysis as described previously [12].

## Results

**Fig. 2:**
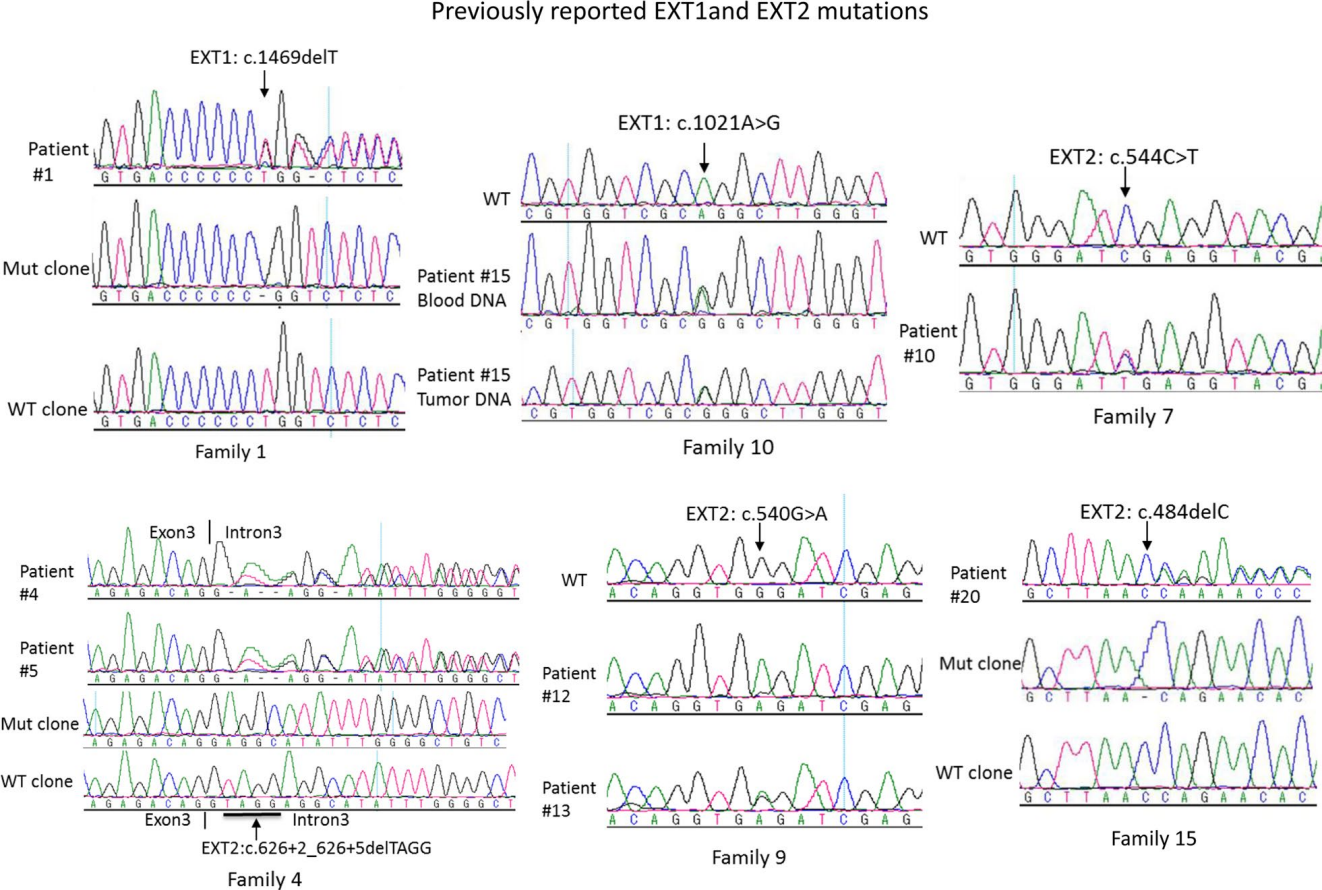
Sequence analysis of *EXT1* and *EXT2* in the patients with hereditary multiple exostoses. Representative electropherograms of previously reported *EXT1*and *EXT2* mutations are shown. Heterozygous mutations are present in the patients and affected family members except for the affected mother (patient#12) in Family 9 who carries a homozygous mutation whereas her daughter (patient#13) has a heterozygous mutation. The mutation is indicated by an arrow

**Fig. 3:**
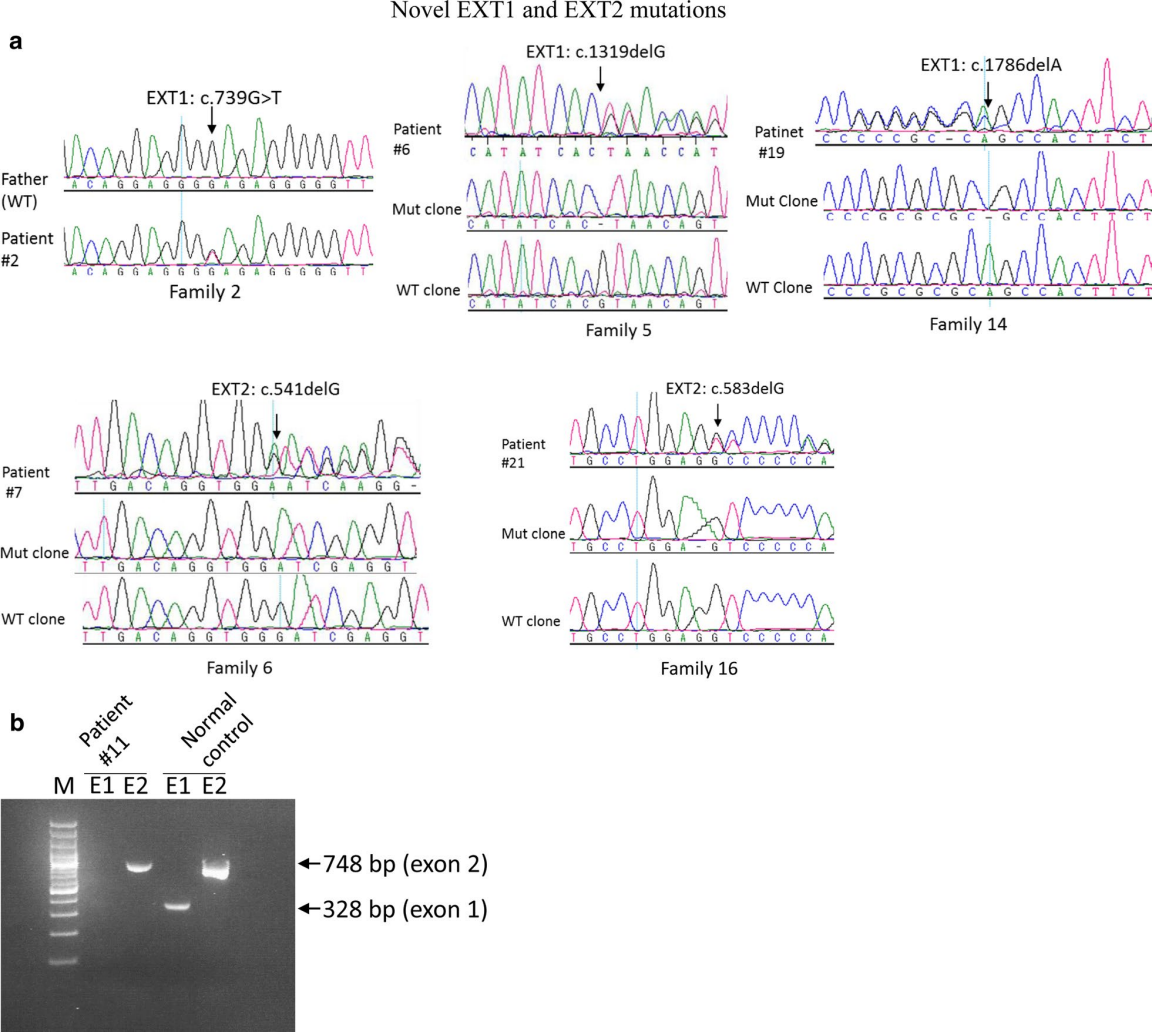
Detection of novel *EXT1* and *EXT2* mutations. **a** Sequence analysis of *EXT1* and *EXT2* in the patients with hereditary multiple exostoses. Representative electropherograms of 5 novel *EXT1* and *EXT2* mutations are shown. They are also de novo mutations except for c.541delG (p.D181Ifs*89) in Family 6. Heterozygous mutations are present only in the patients. The mutation is indicated by an arrow. **b** Agarose gel analysis of a homozygous *EXT2* exon 1 deletion. PCR products were run in a 1.3% agarose gel. Exon 1 was not amplified from patient #11 whereas the remaining exons 2–14 were amplified (only exon 2 amplification was shown)

*EXT1* and *EXT2* mutations were identified in 13 out of 17 (77%) unrelated patients and 18 of total 22 patients (82%) (Table 1). Among them, 7 were *EXT1* mutations including 1 recurrent mutation in one related family member (35%, 6/17 unrelated patients or 32%, 7/22 total patients); 11 were *EXT2* mutations including 4 recurrent mutations from 4 family members (41%, 7/17 unrelated patients, or 50%, 11/22 total patients) (Table 1). Among 13 different mutations, 7 were previously reported mutations (Table 1, Fig. 2) and 6 were novel mutations (Fig. 3). Three novel mutations occurred in the *EXT1*: c.739G > T (p.E247*), c.1319delG (p.R440Lfs*4), and c.1786delA (p.S596Afs*25) and 3 in the *EXT2*: c.541delG (p.D181Ifs*89), c.583delG (p.G195Vfs*75) and a gross homozygous deletion of approximately 10 kb including promoter and exon 1 (Table 1, Fig. 3). In the patient with the homozygous deletion, we were able to amplify exon 2 to 14 successfully, but could not amplify exon 1 and its 5′ untranslated region of about 10 kb, indicating a 10 kb deletion of exon 1 and the promoter region. Five patients from unrelated families were found to have mutations without any family history of the disease and these mutations were thus de novo mutations (29%, 5/17). Interestingly, 4 of them were also novel mutations (Table 1). MLPA analysis was performed to detect large deletions in the patients who had no mutation detected by PCR-sequencing analysis. One large heterozygous deletion involving exons 2–11 was detected (Table 1). Among 13 different mutations, 6 were single nucleotide deletions, 3 were nonsense mutations, 1 missense mutation, 1 splice donor site mutation, and 2 large deletions. Therefore, all the mutations except for one missense mutation (92%, 12/13) are predicted to result in frameshift and truncated proteins devoid of enzymatic activity.

Compared to the patients with *EXT2* mutations, most patients with *EXT1* mutations had more severe phenotype and required surgery. Germline homozygous *EXT2* mutations were identified in two patients (patient #11 and 12 in Table 1) who presented only mild asymptomatic disease and no clinical intervention was required. Furthermore, significant heterogeneity in clinical presentations were demonstrated among family members carrying the same mutations. For example as shown in Table 1, patient#12 carried a homozygous *EXT2* c.540G > A mutation with only mild asymptomatic disease whereas her daughter (patient#13) had a heterozygous *EXT2* c.540G > A mutation and required multiple operations to remove exostosis and correct bone deformity.

## Discussion

In the present study, we have studied *EXT1* and *EXT2* mutation spectrum in 22 patients from 17 unrelated Saudi families. Disease-causing mutations are identified in 77% of patients (13/17) including 6 novel mutations. The frequency of *EXT1* mutation is lower than *EXT2*: 35% (6/17) for *EXT1* and 41% (7/17) for *EXT2*. Twenty-nine percent of patients (5/17) have de novo mutations, which account for 39% (5/13) of mutations identified.

*EXT1* and *EXT2* encode for 746 and 718 amino acids glycosyltransferases, respectively, that are involved in the chain elongation step of heparan sulfate biosynthesis in the cell’s Golgi apparatus [13–15]. Heparan sulfate is an essential component of cell surface and matrix-associated proteoglycans, which function by interacting with key heparin sulfate-binding proteins such as bone morphogenetic proteins (BMPs), fibroblast growth factor (FGF), Hedgehog and Wnt signaling proteins to regulate skeletal growth and morphogenesis [16, 17]. The growth plate of long bones is known to contain large amounts of heparan sulfate proteoglycans, such as syndecan, glypican and perlecan during cartilage development [18]. The glycosyltransferases are ubiquitously expressed type II transmembrane glycoproteins with transmembrane domain at the N-terminal end, an exostosin interaction domain in the center and a catalytic domain at the C-terminal end. EXT1 and EXT2 form a hetero-oligomeric complex in vivo that leads to accumulation of both proteins in the Golgi apparatus. The Golgi-localized EXT1/EXT2 complex possesses substantially higher glycosyltransferase activity than EXT1 or EXT2 alone, suggesting that the hetero-oligomeric complex is the biological form of the enzyme for heparan sulfate biosynthesis and explains mutations in either *EXT1* or *EXT2* gene would result in the loss of enzymatic activity and disease development [19–21].

HME is a rare childhood-onset skeletal disease caused by germline mutations in the tumor suppressor gene *EXT1* or *EXT2*. Most HME patients carry a germline heterozygous loss-of-function mutation in the *EXT1* or *EXT2* and display a 50% reduction of systemic heparin sulfate [22]. It is generally believed that exostosis formation and associated defects, such as growth retardation and skeletal deformities, require loss-of-heterozygosity or a second hit in the affected cells [23, 24]. Mice with single heterozygous deletion of Ext1± or Ext2± are normal. Compound heterozygous Ext1+/−; Ext2+/− deletion mice and conditional Ext1 knockout mice display multiple osteochondromas and closely resemble human HME [25–27]. However, a second hit in the *EXT1* or *EXT2* gene are not common in most cases (more than 60%), suggesting that mechanisms other than *EXT* genetic alterations may play a role in the disease development [28, 29]. In our patients, homozygous germline *EXT2* mutations were detected in two patients (patient #11 and 12 (Table 1, Fig. 2 and 3b). To our knowledge, homozygous germline *EXT1*/*EXT2* mutations have not been reported in the literature. Interestingly, the presence of homozygous germline *EXT2* mutations does not associated with severity of the disease since both patients have mild asymptomatic disease. Furthermore, no significant difference in clinical presentations or disease progression is found between patients with mutation and those without mutation. In fact, significant heterogeneity in disease development and progression are observed among patients with or without mutations. This is even demonstrated among family members carrying the same mutations, indicating epigenetic and/or environmental factors may contribute to the disease development and progression.

It has been reported that *EXT1* mutation is more common (about 65%) than *EXT2* (about 30%) and its protein is less tolerant to the damaging mutations [5, 30]. This may explain *EXT1* mutations usually result in more severe disease phenotype. Indeed, most of our patients with *EXT1* mutations have more severe phenotype and require surgery. In contrast to the higher *EXT1* mutation rate reported in the literature, the frequency of *EXT1* mutation appears to be lower than *EXT2* in our current study. It remains to be determined whether this is due to small sample size or population-specific.

The most common type of mutations in the *EXT1* and *EXT2* genes are inactivating mutations, such as frameshift, nonsense, and splice-site mutations [6, 31, 32]. Based on the HGMD® Professional 2020.1 (Accessed on August 10, 2020), approximately 79% *EXT1* mutations and 75% *EXT2* mutations are inactivating mutations: frameshift 47% (268/566), nonsense 22% (123/566), splice-site 10% (58/565) in the *EXT1*; frameshift 43% (119/278), nonsense 22% (60/278), splice-site 10% (29/278) in the *EXT2*. The remaining *EXT1* mutations are missense (12%, 68/566), gross deletions (7%, 40/566), and complex rearrangements (1%, 7/566) whereas remaining *EXT2* mutations are missense (14%, 40/278) and gross deletions (9%, 26/278). In our current study of Saudi patients, the overall frequency of inactivating *EXT1* and *EXT2* mutations is 92% (12/13): frameshift 46% (6/13), nonsense 23% (3/13), splice-site 8% (1/13), gross deletion (15%, 2/13), which is higher than the overall rate documented in the HGMD (78%, 657/843). This is probably due to small sample size in our study. All of these mutations (92%, 11/12) are predicted to result in truncated proteins devoid of enzymatic activity. Four patients were not found to have *EXT1*/*EXT2* mutations (Patient# 3, 16, 17, 18). Although HME may be confused with enchondroma which is a benign cartilage tumor, enchondroma often affects the cartilage that lines the inside of long bones in the hands and feet. The clinical and radiographic features of our patients (multiple bony outgrowths on the external surface in the metaphysis of long bones) do not support the diagnosis of enchondromas. The involvement of additional genes other than *EXT1*/*EXT2* or other mechanisms may contribute to the disease development [7, 8].

De novo* EXT1* and *EXT2* mutations have been reported to account for approximately 10% of patients [5, 33]. However, higher frequency are reported in other populations: Polish (21%) [34], English (33%) [35], and Chinese (30%) [36]. The high de novo mutation rate in the Saudi patients (29%) indicates that family history should not be relied upon heavily in the diagnosis of the disease.

## Conclusions

We have investigated genetic defects of *EXT1* and *EXT2* in the Saudi HME patients. *EXT1* and *EXT2* mutations are detected in 77% of patients. De novo* EXT1* and *EXT2* mutations are common. The current study further expands the mutation spectrum of HME.

## Data Availability

Data supporting the findings of the study are included in the manuscript.

## Abbreviations

HME: Hereditary Multiple Exostoses
MO: Multiple Osteochondromas
EXT1: Exostosin glycosyltransferase 1
EXT2: Exostosin glycosyltransferase 2
PCR: Polymerase chain reaction
MLPA: Multiplex ligation-dependent probe amplification

## References

[1] Schmale GA, Conrad EU, Raskind WH (1994). The natural history of hereditary multiple exostoses. J Bone Joint Surg Am.

[2] Pacifici M (2017). Hereditary multiple exostoses: new insights into pathogenesis, clinical complications, and potential treatments. Curr Osteoporos Rep.

[3] Czajka CM, DiCaprio MR (2015). What is the proportion of patients with multiple hereditary exostoses who undergo malignant degeneration?. Clin Orthop Relat Res.

[4] Bovée JV (2008). Multiple osteochondromas. Orphanet J Rare Dis.

[5] Jennes I, Pedrini E, Zuntini M, Mordenti M, Balkassmi S, Asteggiano CG, Casey B, Bakker B, Sangiorgi L, Wuyts W (2009). Multiple osteochondromas: mutation update and description of the multiple osteochondromas mutation database (MOdb). Hum Mutat.

[6] Wuyts W, Van Hul W (2000). Molecular basis of multiple exostoses: mutations in the EXT1 and EXT2 genes. Hum Mutat.

[7] Szuhai K, Jennes I, de Jong D, Bovée JV, Wiweger M, Wuyts W, Hogendoorn PC (2011). Tiling resolution array-CGH shows that somatic mosaic deletion of the EXT gene is causative in EXT gene mutation negative multiple osteochondromas patients. Hum Mutat.

[8] Waaijer CJ, Winter MG, Reijnders CM, de Jong D, John Ham S, Bovée JV, Szuhai K (2013). Intronic deletion and duplication proximal of the EXT1 gene: a novel causative mechanism for multiple osteochondromas. Genes Chromosomes Cancer.

[9] Mordenti M, Ferrari E, Pedrini E, Fabbri N, Campanacci L, Muselli M, Sangiorgi L (2013). Validation of a new multiple osteochondromas classification through Switching Neural Networks. Am J Med Genet A.

[10] Cebeci AN, Zou M, BinEssa HA, Alzahrani AS, Al-Rijjal RA, Al-Enezi AF, Al-Mohanna FA, Cavalier E, Meyer BF, Shi Y: Mutation of SGK3, a novel regulator of renal phosphate transport, causes autosomal dominant hypophosphatemic rickets. *J Clin Endocrinol Metab* 2020, 105(6). 10.1210/clinem/dgz260 31821448

[11] Sarrión P, Sangorrin A, Urreizti R, Delgado A, Artuch R, Martorell L, Armstrong J, Anton J, Torner F, Vilaseca MA (2013). Mutations in the EXT1 and EXT2 genes in Spanish patients with multiple osteochondromas. Sci Rep.

[12] Raef H, Zou M, Baitei EY, Al-Rijjal RA, Kaya N, Al-Hamed M, Monies D, Abu-Dheim NN, Al-Hindi H, Al-Ghamdi MH (2011). A novel deletion of the MEN1 gene in a large family of multiple endocrine neoplasia type 1 (MEN1) with aggressive phenotype. Clin Endocrinol (Oxf).

[13] Nadanaka S, Kitagawa H (2008). Heparan sulphate biosynthesis and disease. J Biochem.

[14] Pacifici M (2018). The pathogenic roles of heparan sulfate deficiency in hereditary multiple exostoses. Matrix Biol.

[15] Lind T, Tufaro F, McCormick C, Lindahl U, Lidholt K (1998). The putative tumor suppressors EXT1 and EXT2 are glycosyltransferases required for the biosynthesis of heparan sulfate. J Biol Chem.

[16] Nagarajan A, Malvi P, Wajapeyee N (2018). Heparan sulfate and heparan sulfate proteoglycans in cancer initiation and progression. Front Endocrinol (Lausanne).

[17] Huegel J, Sgariglia F, Enomoto-Iwamoto M, Koyama E, Dormans JP, Pacifici M (2013). Heparan sulfate in skeletal development, growth, and pathology: the case of hereditary multiple exostoses. Dev Dyn.

[18] Farach-Carson MC, Hecht JT, Carson DD (2005). Heparan sulfate proteoglycans: key players in cartilage biology. Crit Rev Eukaryot Gene Expr.

[19] Busse M, Feta A, Presto J, Wilén M, Grønning M, Kjellén L, Kusche-Gullberg M (2007). Contribution of EXT1, EXT2, and EXTL3 to heparan sulfate chain elongation. J Biol Chem.

[20] McCormick C, Duncan G, Goutsos KT, Tufaro F (2000). The putative tumor suppressors EXT1 and EXT2 form a stable complex that accumulates in the Golgi apparatus and catalyzes the synthesis of heparan sulfate. Proc Natl Acad Sci USA.

[21] Senay C, Lind T, Muguruma K, Tone Y, Kitagawa H, Sugahara K, Lidholt K, Lindahl U, Kusche-Gullberg M (2000). The EXT1/EXT2 tumor suppressors: catalytic activities and role in heparan sulfate biosynthesis. EMBO Rep.

[22] Anower EKMF, Matsumoto K, Habuchi H, Morita H, Yokochi T, Shimizu K, Kimata K (2013). Glycosaminoglycans in the blood of hereditary multiple exostoses patients: half reduction of heparan sulfate to chondroitin sulfate ratio and the possible diagnostic application. Glycobiology.

[23] Reijnders CM, Waaijer CJ, Hamilton A, Buddingh EP, Dijkstra SP, Ham J, Bakker E, Szuhai K, Karperien M, Hogendoorn PC (2010). No haploinsufficiency but loss of heterozygosity for EXT in multiple osteochondromas. Am J Pathol.

[24] Hameetman L, Szuhai K, Yavas A, Knijnenburg J, van Duin M, van Dekken H, Taminiau AH, Cleton-Jansen AM, Bovee JV, Hogendoorn PC (2007). The role of EXT1 in nonhereditary osteochondroma: identification of homozygous deletions. J Natl Cancer Inst.

[25] Jones KB, Piombo V, Searby C, Kurriger G, Yang B, Grabellus F, Roughley PJ, Morcuende JA, Buckwalter JA, Capecchi MR (2010). A mouse model of osteochondromagenesis from clonal inactivation of Ext1 in chondrocytes. Proc Natl Acad Sci USA.

[26] Matsumoto Y, Matsumoto K, Irie F, Fukushi J, Stallcup WB, Yamaguchi Y (2010). Conditional ablation of the heparan sulfate-synthesizing enzyme Ext1 leads to dysregulation of bone morphogenic protein signaling and severe skeletal defects. J Biol Chem.

[27] Sgariglia F, Candela ME, Huegel J, Jacenko O, Koyama E, Yamaguchi Y, Pacifici M, Enomoto-Iwamoto M (2013). Epiphyseal abnormalities, trabecular bone loss and articular chondrocyte hypertrophy develop in the long bones of postnatal Ext1-deficient mice. Bone.

[28] Zuntini M, Pedrini E, Parra A, Sgariglia F, Gentile FV, Pandolfi M, Alberghini M, Sangiorgi L (2010). Genetic models of osteochondroma onset and neoplastic progression: evidence for mechanisms alternative to EXT genes inactivation. Oncogene.

[29] de Andrea CE, Reijnders CM, Kroon HM, de Jong D, Hogendoorn PC, Szuhai K, Bovée JV (2012). Secondary peripheral chondrosarcoma evolving from osteochondroma as a result of outgrowth of cells with functional EXT. Oncogene.

[30] Cousminer DL, Arkader A, Voight BF, Pacifici M, Grant SFA (2016). Assessing the general population frequency of rare coding variants in the EXT1 and EXT2 genes previously implicated in hereditary multiple exostoses. Bone.

[31] Ishimaru D, Gotoh M, Takayama S, Kosaki R, Matsumoto Y, Narimatsu H, Sato T, Kimata K, Akiyama H, Shimizu K *et al*: Large-scale mutational analysis in the EXT1 and EXT2 genes for Japanese patients with multiple osteochondromas. *BMC Genet* 2016, 17:52. doi:10.1186/s12863-12016-10359-12864. 10.1186/s12863-016-0359-4 PMC4784393 26961984

[32] Sarrion P, Sangorrin A, Urreizti R, Delgado A, Artuch R, Martorell L, Armstrong J, Anton J, Torner F, Vilaseca MA *et al*. Mutations in the EXT1 and EXT2 genes in Spanish patients with multiple osteochondromas. *Sci Rep* 2013, 3:1346. doi:10.1038/srep01346. 10.1038/srep01346 PMC3581825 23439489

[33] Ciavarella M, Coco M, Baorda F, Stanziale P, Chetta M, Bisceglia L, Palumbo P, Bengala M, Raiteri P, Silengo M (2013). 20 novel point mutations and one large deletion in EXT1 and EXT2 genes: report of diagnostic screening in a large Italian cohort of patients affected by hereditary multiple exostosis. Gene.

[34] Jamsheer A, Socha M, Sowińska-Seidler A, Telega K, Trzeciak T, Latos-Bieleńska A (2014). Mutational screening of EXT1 and EXT2 genes in Polish patients with hereditary multiple exostoses. J Appl Genet.

[35] Philippe C, Porter DE, Emerton ME, Wells DE, Simpson AH, Monaco AP (1997). Mutation screening of the EXT1 and EXT2 genes in patients with hereditary multiple exostoses. Am J Hum Genet.

[36] Kang QL, Xu J, Zhang Z, He JW, Fu WZ, Zhang ZL (2013). Mutation screening for the EXT1 and EXT2 genes in Chinese patients with multiple osteochondromas. Arch Med Res.

[37] Ahn J, Ludecke HJ, Lindow S, Horton WA, Lee B, Wagner MJ, Horsthemke B, Wells DE (1995). Cloning of the putative tumour suppressor gene for hereditary multiple exostoses (EXT1). Nat Genet.

[38] Li Y, Wang J, Wang Z, Tang J, Yu T (2017). A genotype-phenotype study of hereditary multiple exostoses in forty-six Chinese patients. BMC Med Genet.

[39] Dobson-Stone C, Cox RD, Lonie L, Southam L, Fraser M, Wise C, Bernier F, Hodgson S, Porter DE, Simpson AH (2000). Comparison of fluorescent single-strand conformation polymorphism analysis and denaturing high-performance liquid chromatography for detection of EXT1 and EXT2 mutations in hereditary multiple exostoses. Eur J Hum Genet.

[40] Jennes I, Entius MM, Van Hul E, Parra A, Sangiorgi L, Wuyts W (2008). Mutation screening of EXT1 and EXT2 by denaturing high-performance liquid chromatography, direct sequencing analysis, fluorescence in situ hybridization, and a new multiplex ligation-dependent probe amplification probe set in patients with multiple osteochondromas. J Mol Diagn.

[41] Jennes I, de Jong D, Mees K, Hogendoorn PC, Szuhai K, Wuyts W: Breakpoint characterization of large deletions in EXT1 or EXT2 in 10 multiple osteochondromas families. *BMC Med Genet* 2011, 12:85 doi:10.1186/1471-2350-1112-1185. 10.1186/1471-2350-12-85 PMC3152881 21703028

